# 743. Efficacy and persistence of 3M™ Skin and Nasal Antiseptic for reduction of *S. aureus* colonization *ex vivo* porcine tissue model

**DOI:** 10.1093/ofid/ofad500.804

**Published:** 2023-11-27

**Authors:** Isabel A Laubach, Marnie L Peterson, Ranjani Parthasarathy

**Affiliations:** Perfectus Biomed Group now part of NAMSA, Jackson, Wyoming; Perfectus Biomed Group now part of NAMSA, Jackson, Wyoming; 3M, St. Paul, Minnesota

## Abstract

**Background:**

Surgical-site infections can result from nasal colonization of *Staphylococcus aureus* prior to surgery. Nasal decolonization with iodine-based products is a common way to combat this problem. The efficacy and persistence of nasal decolonization products is a crucial component for this presurgical protocol to be successful. An ex vivo porcine mucosal model allows for clinically relevant testing of formulations without the need for live animals.

**Methods:**

Fresh porcine vaginal mucosa was processed to create 5 mm diameter explants. One group was left uninfected, and the other was infected with prepared methicillin-resistant *S. aureus* (MRSA) Xen30 to achieve 10^6^ CFU/explant and incubated for 2h at 37 °C. After 2-hour incubation, explants were treated with formulation for 1h then gently washed with mucin and incubated for 24h at 37 °C. Uninfected explants were photographed prior to treatment, immediately post treatment, pre and post wash, and at hour intervals following mucin wash. Infected explants were collected into neutralizing solution at 24h, vortexed and sonicated to liberate bacteria then plated to enumerate CFU/explant. Additional infected explants were stained with LIVE/DEAD stain and imaged by fluorescence microscopy at 24h.

**Results:**

Pictures of uninfected, treated explants showed that 3M™ Skin and Nasal Antiseptic (SNA) formulation persisted visually on the tissue up to 6h post wash while Betadine was no longer visible on tissue at 1h post wash. At 24h post wash, explants treated with Skin and Nasal had no recoverable colony forming units while explants treated with Betadine had regrown to ∼7 Log_10_ CFU/explant. This finding was confirmed by staining and fluorescent imaging. Fluorescent imaging showed that treatment of uninfected explants with SNA or Betadine did not compromise tissue viability compared to untreated control; however, infected explants treated with Skin and Nasal retained the most tissue viability.

Fluorescent images of porcine vaginal mucosa stained with LIVE/DEAD stain following 24h incubation with formulations.

**Figure:**
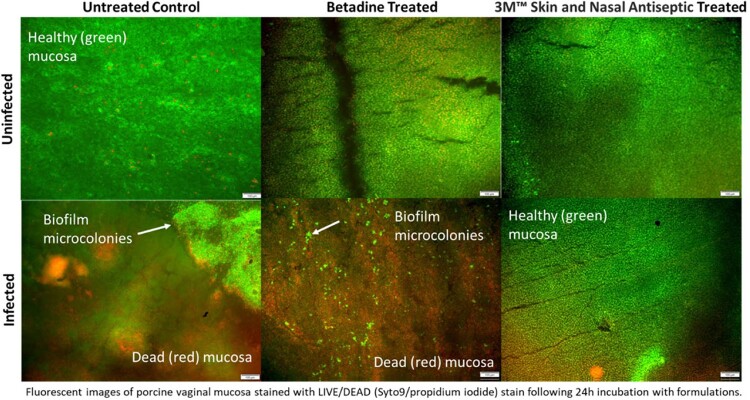

**Conclusion:**

The material properties of SNA prep allow it to remain on the tissue even after washing resulting in longer lasting activity against MRSA Xen30 than Betadine treatment over 24 hours. By persisting, the SNA treated explants showed reduced tissue damage by MRSA than was observed in the Betadine and untreated groups.

**Disclosures:**

**Ranjani Parthasarathy, PhD**, 3M: Have patents|3M: 3M employee|3M: Stocks/Bonds

